# The influence of parents’ perception on online education and training brand recognition

**DOI:** 10.3389/fpsyg.2022.989401

**Published:** 2022-09-01

**Authors:** Biyun Xue, Ye Song

**Affiliations:** ^1^School of Music and Dance, Henan Normal University, Xinxiang, China; ^2^School of Marxism, Henan Normal University, Xinxiang, China; ^3^Faculty of Education, Henan Normal University, Xinxiang, China

**Keywords:** parental perception, online education and training, brand recognition, online educational platform, business models

## Abstract

At present, the academic education of Chinese students is basically public education, but the quality training is mainly handed over to the market for training. Therefore, China’s online education and training institutions have gradually developed under this demand. With the improvement of people’s living standards, families have higher and higher requirements for children’s education, expecting that children can be well improved in physical, mental and psychological aspects, and hoping that they will have their own advantages in the future competition. Therefore, this paper studies the influence of parents’ perception on the brand recognition of online education and training. Through the analysis of dance online training institutions, the research shows that among the three categories, teachers’ Graduation schools account for the highest proportion, with an average proportion of 47.07%, followed by teachers’ grade certificates, with a proportion of 32.29%, and teachers’ competition scores of 25.57%. Therefore, in the process of operation, online dance training institutions should meet the needs of parents to understand the professional level of teachers. Improve the service system of training institutions, improve the parent brand recognition and the number of customers of training institutions, further improve online education and training institutions, and provide them with improvement suggestions and measures for reference.

## Introduction

With the continuous improvement of the quality of education in China, students’ parents are paying more attention to education, so their requirements for education are becoming higher and higher. At present, the academic education of Chinese students is basically public education, but the quality training is mainly handed over to the market for training. Therefore, China’s online education and training institutions have gradually developed under this demand (Cho, 2019). Promoting the connotative development of online education is a new trend in the development of China’s education industry and a key direction to improve the quality of education. Therefore, this paper studies the brand recognition degree of dance online training (Yang, 2019). In order to improve its own quality, strengthen the brand construction, and enhance its own reputation in the education market, so as to obtain growth benefits, the current dance online education and training is an important means for Chinese Dance online education institutions to improve their own level (Filonenko and Ushakova, 2020). Some dance online education and training institutions have some problems, such as low training quality, chaotic fees, boring teaching content, difficult to guarantee the strength and level of teachers, and difficult to meet the needs of consumers. In order to better attract students, when teachers teach dance online, they create opportunities for students to learn new knowledge and show more content that students are interested in. For example, students now like the handsome jazz dance (Nyamupanemunda and Deshinta, 2022). Although there may be no special Jazz practice items in our textbooks, teachers can still play some relevant videos to students to attract their attention. Because the brand represents the core competitiveness of the enterprise and the value embodiment of the excellent product quality of the enterprise. This paper combines the relevant theories and senior opinions of dance online training institution brand and education brand, refines the theoretical essence and successful practical experience, comprehensively uses the research methods of literature research, analysis, questionnaire survey and so on, studies the brand construction of online education and training, and is committed to solving the problem of how the institution carries out brand construction as a new small and medium-sized training institution (Ashraf et al., 2021). Dance online training institutions can also carry out parent-child project evenings, so that they can participate in them together, so that parents and students can have more opportunities to contact the project together and feel the changes brought about by the training. Training institutions can also take this opportunity to show their superior professional level and satisfactory service attitude. So as to stimulate parents’ expectations, form greater project learning needs and improve recognition. Improve brand familiarity of educational institutions through continuous display to establish brand awareness of educational institutions. Once a rich brand awareness of educational institutions is established, the focus of enrollment marketing can be placed on shaping the brand image of educational institutions. The matching method between the brand of educational institutions and categories (such as advertising slogans) determines the strength of the connection of educational categories. Make students and parents experience any elements of the brand name, symbol, trademark, characteristics, packaging, or slogan of educational institutions Can improve people’s familiarity with the brand elements of educational institutions and their popularity. In addition, the more brand elements of educational institutions that can be strengthened, the better.

People’s consumption concept and demand have also changed greatly with the development of economy. With the improvement of living standards, families have higher and higher requirements for children’s education, expecting that children can be well improved in physical, mental and psychological aspects, and hoping that they will have their own advantages in the future competition. Therefore, this paper studies the online education and training brand from the perspective of parents’ perception. Choosing a high-quality dance online education institution is a primary factor for parents to consider in the process of cultivating their children’s comprehensive quality education (Iuliana et al., 2018). The design focus of the brand is to directly interpret the brand emotion through the form, color and functional characteristics of the brand image. It is closely related to the general impression of the public’s long-term use of a brand, and is also affected by personal cognition, thought, experience, cultural background and other factors. Brand design is more about improving emotional ability. Through design, children’s self cognitive ability is improved to a certain extent, and a long-term emotional bond between children and the brand is established. Therefore, children’s brand design should not only meet the basic functions and aesthetic needs, but also meet the emotional needs of users. Due to the importance of parents’ training their children in the society, various professional educational institutions have emerged at the right time and have fully penetrated into the family life under the Chinese educational background. These problems will directly affect parents’ expectations for their children’s growth and recognition of training institutions, and indirectly affect the survival and development of online education training institutions (Mcdevitt, 2021). The brand recognition of students’ parents affects the development of online education institutions. Considering the students’ limited ability of expression and understanding, parents are the direct decision-makers in choosing training institutions (Rahman et al., 2020). Therefore, in this paper, with the recognition degree of students’ parents as the core, with the help of investigating the brand recognition degree of students’ parents to dance online education and training institutions, an investigation is carried out to optimize the training institutions on this basis, which is indispensable to the sustainable development of dance online education and training institutions (Vega and Eppendi, 2021).

With the increase of the number of trainees, enterprises have exposed such problems as the lack of standards for service products, the difficulty in ensuring the strength and level of teachers, and the difficulty in meeting the needs of consumers, which make the further growth of these enterprises slow. Through the data analysis of the survey results on the brand recognition degree of the teaching parents of children’s dance online education and training institutions, this paper puts forward strategies for the teaching of children’s sports dance training institutions in terms of low satisfaction, and finally achieves the goal of improving the brand recognition degree of the teaching parents. For example, there is no research on the online education and training institutions from the perspective of parents’ brand recognition degree (Syamsuri, 2020). Therefore, this research has certain theoretical significance. The research on parents’ satisfaction can improve the fault-tolerant rate of dance online education and training institutions in the market development, and provide corresponding theoretical suggestions for their long-term development. As a part of the main body of market education, dance online education and training institutions can maintain a sound development only if they meet the expectations of parents for their children to sign up for training. Therefore, in the fierce competitive environment, training institutions need to focus on improving parents’ recognition of the brand and attracting more parents to sign up, so as to gain a firm foothold in the training market and achieve the goal of continuous development and expansion (Winkelmann and Eberman, 2020). Research on the brand recognition degree of the students’ parents of dance online education and training institutions, and then draw the factors affecting the satisfaction of parents, so as to summarize the strategies that can be adopted for the future sustainable development, improve the service system of the training institutions, improve the parent brand recognition degree and the number of customers of the training institutions, further improve the dance online education and training institutions, and provide them with improvement suggestions and measures for reference (Elmunsyah et al., 2020).

The innovative contribution of this paper lies in the construction of an online dance teaching system. Brand design is more about improving emotional ability. Through design, children’s self cognition ability is improved to a certain extent, and a long-term emotional bond is established between children and brands. Therefore, brand design should not only meet the basic functions and aesthetic needs, but also meet the emotional needs of users. How to effectively use the rich brand teaching resources on the Internet is the key problem to be solved at present. The construction of online dance teaching system can enable students to view learning content at any time, make full use of Internet resources and scattered time around the computer environment, and improve learning efficiency. Consolidate learning achievements, expand learning influence, lay a solid foundation for better serving the society, and develop and carry forward dance in the future. Combined with curriculum learning, it is conducive to students’ autonomous learning potential, and also provides a shortcut for inter school communication and curriculum resource sharing.

The overall structure of this paper consists of five parts.

The first chapter introduces the background and significance of online education and training brand, and then introduces the main work of this paper. The second chapter mainly introduces the related work of online education and training brand and the research content of online education and training brand based on the method proposed in this paper. The third chapter introduces the research of parents’ perception on online education and training, and puts forward the teaching mode of dance online training institutions and the teacher-student interaction subsystem. The fourth chapter analyzes the experimental part. The fifth chapter is a summary of the full text.

## Related work

In terms of the elements of business model, most online education and training institutions do not have the ability to master key resources, and they are faced with the problems of high pressure of marketing funds, low liquidity of users, low renewal rate of users, and low stickiness of users, so they cannot achieve long-term profits. It is precisely because the business model of online educational institutions is not clear, and when choosing the business model, it doesn’t aim at creating value for customers, so that the goods provided by them can’t meet the needs of users, which leads to the above problems. Therefore, many scholars have carried out research on this issue.

### Research on online education and training

Teachers lacked basic training and physical training in the process of online physical education dance teaching for children, and often ignored teaching rules, which led to the best training effect (Aribarg and Schwartz, 2020). In the process of online sports dance training and education, the training duration is not enough, and the sports dance curriculum system needs to be improved (Aribarg and Schwartz, 2020). The number of male students is obviously lower than that of female students, which affects the learning effect. Institutions pay too much attention to economic benefits and neglect the investment of publicity and other activities (Westera et al., 2018). The marketing war has increased the cost of customer acquisition of educational institutions, but the realization rate of users has not reached the expectation of marketing activities. Many users choose to buy fixed price courses, but they are not satisfied with the course results. They decided not to continue to buy courses, resulting in a survival rate of only 70% in most educational institutions, which increased the operating costs of enterprises (Park and Holloway, 2018). At present, the effectiveness of the traditional brand promotion model is affected. Education and training institutions can make use of new media, big data and Internet media resources, pay attention to the combination of big events and networks, information feedback between enterprises and the public, and interactive two-way communication mechanism. Online promotion and offline activities should be carried out simultaneously to vigorously mobilize the participation and enthusiasm of stakeholders and improve the efficiency and effect of education brand promotion (Nikitas et al., 2019). Through the investigation of online Latin dance training institutions for teenagers in Qingdao, it is believed that the combination of theoretical research and practice should be strengthened in online sports dance training, and training institutions should pay more attention to the standardization of teaching routines and the standards of technical movements in the training process. Meanwhile, relevant associations and competent departments should strengthen the guidance and management of online sports dance training institutions (Cheong et al., 2018). With the rapid development of online education industry, a lot of money has been invested in this industry. From 2011 to 2016, capital poured into the online education industry. However, in 2017, the online education industry generally suffered losses, and capital gradually returned to rationality (Hu, 2019). Through the investigation and analysis of online Latin dance training and education institutions for children in urban areas of Xi’an, it is known that online sports dance training and education institutions for children in Xi’an are mostly private enterprises, with weak teachers and part-time teachers. The lack of stability of the teaching team affects the realization of teaching objectives, and the training methods are too adult, which affects children’s learning effect (Montes and Montes, 2021). From the enterprise level, during the epidemic prevention and control period in the first half of 2020, the Ministry of Education put forward the call of “stopping classes and studying,” which enabled consumers to better accept online education on the Internet and brought user traffic to online education enterprises. However, in the post-epidemic period, it is necessary to analyze whether the current business model of the enterprise is reasonable, whether it can meet the needs of users and achieve sustained profitability, how to convert the traffic into its own stable users and how to better enhance the user stickiness (Duman, 2018). To better optimize the marketing strategy of online education platform for children’s English, we must start with users’ acceptance behavior, so as to identify the key factors that affect users’ acceptance behavior, and then further expand the platform in different regions. “Research on the development of online sports dance education for children in Heilongjiang Province” pointed out that although the sports dance projects in Heilongjiang Province have developed well. However, there are still some problems in the process of online training. For example, children’s sports dance teachers lack knowledge and are too traditional online dance teaching methods and means. Some students and parents are not well aware of the positive impact of sports dance (Jinapor and Larbi, 2020).

### The research methods proposed in this paper

Drawing on the results of previous studies, this paper studies the impact of online education and training brand recognition from the perspective of parents’ perception, and uses the theory of parents’ recognition to design the evaluation standards, models and systems of online education and training. Provide suggestions for the healthy development of industry norms by investigating the recognition degree and improving the service quality of online education and training institutions. Parents’ recognition of online education and training brands can not only reflect the operation quality of online education and training institutions, but also have a good feeling and predict the future development trend. For online education and training institutions, a better degree of parental recognition can bring better operational feedback and support the long-term development of online education and training institutions. As the service port of the online education and training service industry, online education and training institutions should not only carry out education from the perspective of educating people, but also implement the principle of service first to improve the economic value of their own industry. At the same time, in combination with local conditions, they should implement standardized service models to promote parents’ positive evaluation of their own institutions and improve their recognition. Only in this way can they get higher market recognition and development prospects.

## Research on parents’ perception of online education and training

Network education has also absorbed the advantages of traditional education in the tortuous process. The original ideal mode has been abandoned in the aspects of examination organization, courseware design, teaching interaction and so on. More and more close to traditional education. In addition, online education researchers are more and more deeply aware of the importance of emotional communication in traditional education. And try to integrate teachers and learners into a virtual classroom through various technical means to make up for the lack of emotional education and humanistic education. In the training, parents have increased their knowledge through the platform of network. Increased awareness and strengthened awareness. It is agreed that the opening of parent classes is of great significance to the growth of children. Under the external conditions of the market economy, many online education and training institutions’ school running ideas and courses are more in line with the market demand, with richer cultural connotation, more comfortable hardware facilities and environmental atmosphere, showing a development trend of flexible school running and management, distinctive characteristics, and continuous improvement of internal quality and quality. In the Internet era, the dance training industry is inevitably affected. Where and how big the impact is, and what are the advantages and disadvantages of the industry, deserve our in-depth study. Therefore, from the perspective of parents’ perception, this paper studies dance online education and training.

### Teaching mode of dance online training institutions

In the process of preschool education, the design of professional dance courses should be comprehensively explored from the perspective of combining online and offline. This kind of blended teaching has more advantages, can improve the overall teaching quality, and can better integrate students’ learning time (Karaman and Efilti, 2019). There is very little time limit for online teaching, and students can splice a lot of fragmented time to start learning. Moreover, in the process of online learning, there is also very little space limit, and students’ enthusiasm for learning will be more mobilized. Online dance teaching can break through the difficulties existing in traditional teaching. The main value activities of the basic value chain of online dance training institutions include seven parts: publicity, enrollment, payment, teaching, feedback communication after class, competition or final class performance, and advanced training. Auxiliary value activities include infrastructure management, human resource management, curriculum management, brand management and comprehensive management. Dance and music is the triple teaching of “dance and music,” “dance and singing,” and “dance and practice.” For the traditional offline teaching, there are many difficulties in this triple teaching. Teachers can only carry out rigid exercises, which not only dampen students’ learning enthusiasm, but also inhibit their creative desire and imagination (Ram et al., 2020). However, with the help of words, music and videos of new media, teachers can set special teaching situations so that students can better perceive dance and music. In addition, in online teaching, teachers can help students grasp the overall rhythm through multimedia and their own guidance. Because of the characteristics of dance itself, teachers mainly adopt the teaching mode of “transmission-reception.” Teachers directly impart dance knowledge and basic skills to students, and students mainly acquire knowledge and skills by listening to lectures and doing exercises. Dance training institutions should comprehensively consider the external environmental factors of the institutions in the early stage of site selection planning, including convenient parking, shopping and entertainment around, security around and convenient transportation. Pay attention to the construction of internal environment, including safety conditions and sanitary conditions. In the training activities of dance training institutions, more attention is paid to the education and training activities, staying in the classroom teaching process, thus ignoring the cultural value of project training. Strengthening the spread of sports dance culture can make parents get a higher degree of brand recognition. In the survey of dance training institutions, it was found that some dance teachers did not teach on the basis of teaching materials. Even the dance teaching materials used by some teachers are unreasonable. In teaching, we did not start from the actual level of dance, and the teaching content tends to be adult or too professional. Neglecting the ability to understand and digest dance skills is not conducive to quickly and accurately grasp dance movements, resulting in the effect of dance training is not obvious.

**FIGURE 1:**
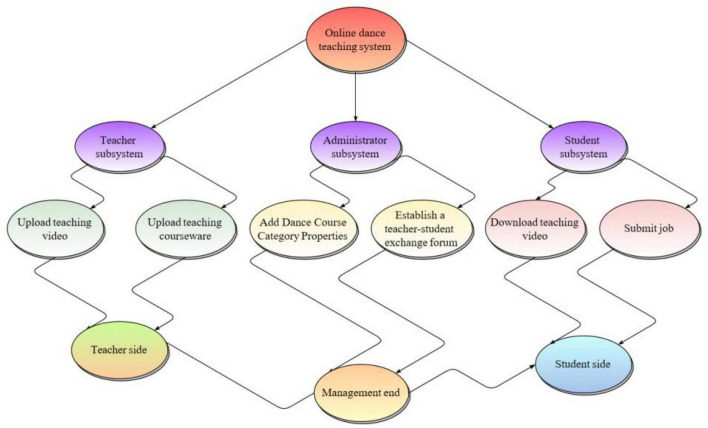
Function of sub module of online dance teaching system.

Dance is a skill-based course. In the process of learning, every movement and every look needs teachers’ demonstration and guidance. Students begin with basic imitation learning, and through teachers’ constant correction and guidance, they complete their own flesh-and-blood memory, and gradually master the key points and skills of every movement, thus realizing their learning goals (Hakyemez et al., 2018). As the goal of dance teaching curriculum is to train students to learn dance knowledge and demonstrate dance skills, and it is also a key course for dance students to exercise their temperament and shape their good bodies, how to make effective use of online rich teaching resources is a key problem to be solved at present. Constructing a distance online learning system can enable students to check the learning content at any time, make full use of Internet resources and the fragmented time in the computer surrounding environment, improve learning efficiency, consolidate learning achievements, expand learning influence, and lay a solid foundation for better serving the society and developing and carrying forward the dance cause in the future. Therefore, this paper constructs an online dance teaching system, as shown in Figure 1.

In online teaching, teachers can not only upload the teaching videos in dance and music classes to various network platforms for students to watch, think and practice, but also make an appointment with parents to conduct live teaching on the network live broadcasting platform, providing a broader stage for students to learn dance and music independently (Rodideal, 2020). The final lifting loss function of the system mainly includes three loss functions, including confrontation loss, feature matching loss, and perception loss, as shown in the formula


(1) $$\min\left(\sum_{k=1}^{a}\lambda \,\left(G,D_{k}\right)\right)$$

The first term of the loss function represents the multi task discrimination loss: $\sum_{k=1}^{a}\lambda \,\left(G,D_{k}\right)$. And the loss function in each task is adversarial loss.

The second term is the loss of feature matching. Feature matching is performed on the feature maps of all layers in the discriminator network. Feature extraction and matching consists of three steps: key point detection, key point feature description, and key point matching. A feature is a piece of information related to solving a computing task related to an application. Features may be specific structures in the image, such as points, edges, or objects. The key descriptor is a vector used to describe the image patch value around the key. The description methods include the methods that compare the original pixel values and more complex methods, such as the histogram of gradient direction. The specific loss expression is shown in the formula


(2) $$L_{e\,a\,t\,M\,a\,t\,e\,h}\,\left(G,D_{k}\right)=E_{g,x}$$

Where *k* represents the discriminator network, *g* represents the *g* layer in the discriminator network, and *D* represents the picture waiting to be generated.

Update the discriminator parameters to maximize the following functions


(3) $$\upsilon =\sum_{k=1,2,3}L_{g\,a\,n}$$

(4) $$\theta_{d}\leftarrow \theta_{d}+\eta \,\Delta \,\left(\theta_{d}\right)$$

Update the generator parameters to minimize the following functions


(5) $$\upsilon =\sum_{k=1,2,3,}L_{g\,a\,n}\,\left(G,D_{k}\right)$$

Thus, the calculation between the real dance image *x* and the generated image *w* is as follows


(6) $$d^{2}=\left(m,c\right),\left(m_{w},c_{w}\right)$$

Where *d*^2^ is the tracing operation, that is, summing all diagonal elements. In particular, a lower value means better dance image quality and diversity.

Next, we will introduce the part of loss value decreasing during training. In the process of online teaching, teachers can’t find the problems existing in students’ practice in time, nor can they guide and demonstrate hand in hand. Even in online live courses, the interaction between teachers and students is blocked by the screen, which can’t achieve the effect of face-to-face correction, thus limiting the development of online teaching (Novianti and Garzia, 2020).

### Teacher student interaction subsystem

**FIGURE 2:**
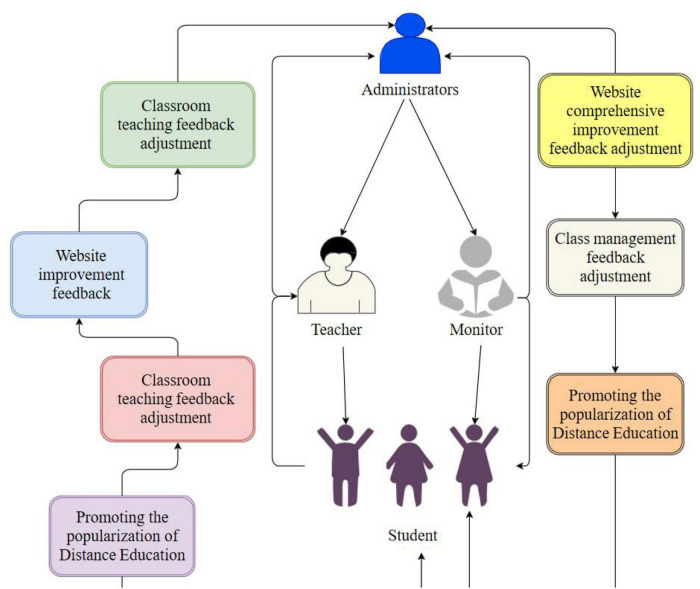
Relationship distribution.

Through positive motivation, general administrators can actively let teachers and squad leaders have an impact on students, and teachers and squad leaders further affect students, which is called “positive impact.” The impact of general teachers or squad leaders on administrators, or students’ impact on teachers and squad leaders, or students’ impact on administrators is called “negative feedback.” General negative feedback can provide a certain positive impact on the normal development of their work. In the classroom, teachers can also use different applications to complete online interaction such as asking questions, and guide students’ initiative and self-consciousness in class, so as to better complete classroom teaching. In the process of classroom teaching, teachers can also divide different teaching contents into different links, set different checkpoints, enhance students’ interest in learning, and help students improve their ability to solve difficulties. The sharing of curriculum resources among teachers in the school enables students to enjoy more high-quality teaching resources. The sharing of possible contents between teachers and students, including problem discussion, mid-term and final exams, and homework mutual evaluation, is integrated with curriculum learning, which is conducive to students’ autonomous learning potential, and also provides a shortcut for inter school exchange and sharing of curriculum resources. The distribution of the relationship among teachers, administrators, monitor and students is shown in Figure 2.

Similarly, for teachers, if they feel that uploading video is inefficient, they can upload action images first, and then upload the whole relevant video, so as to maximize the interactivity of the website and between teachers and students. Basically, from the administrator to each link, there is corresponding feedback adjustment, and each time it is propagated downward, the lower level goal will carry out negative feedback adjustment on the sub goal, which will play a certain incentive role for both teaching and management. The teacher should first demonstrate to the students, explain the basic essentials of the action to the students, and try to be more detailed to facilitate the students’ understanding. Then, more time will be allocated to the students themselves to summarize the exercises and improve their own abilities. The calculation process of forward propagation is


(7) $$s_{t}=\tanh\left(U\,x+W\,s_{t-1}\right)$$

(8) $$o_{t}=s\,o\,f\,t\,\max\left(V\,s_{t}\right)$$

The calculation formula is as follows


(9) $$s\,o\,f\,t\,\max\left(z\right)=\frac{e^{z\,j}}{\sum_{k=1}e^{z\,k}}$$

The expression of the active function is


(10) $$S\,i\,g\,m\,o\,i\,d=\sigma \,\left(x\right)=\frac{1}{1+e^{-x}}$$

The gradient vanishing problem may occur when the back propagation relies on the gradient to update the network parameters


(11) $$T\,a\,n\,h\,\left(x\right)=\frac{\sinh\left(x\right)}{\cosh\left(x\right)}=\frac{e^{x}-e^{-x}}{e^{x}}$$

How much previous state to keep and how much information to discard


(12) $$f_{t}=\sigma \,\left(W_{f}\,\left[h_{t-1},x_{t}\right]\right)$$

By reading the output *h*_*t–1*_ of the previous time and the input *x*_*t*_ of the current time *t*, it is transformed into a number between 0 and 1 through the sigmoid activation function.

A new candidate value vector *C*_*t*_ is calculated and added to the cell state


(13) $$i_{t}=\sigma \,\left(W_{i}\cdot \left[h_{t-1},x_{t}\right]\right)$$

(14) $$C_{t}=\tanh\left(W_{c}\cdot \left[h_{t-1},x_{t}\right]+b_{c}\right)$$

Because each student has a different perception of dance, and some students have been exposed to dance in the original period, while some students are a blank sheet of paper and know nothing. The differences between students are uneven. At present, the online teaching design of dance courses for most preschool education majors is carried out under the guidance of the teaching concept of “flipped classroom,” which mainly reflects the progressiveness and scientificity of the design from the following aspects. Teachers can assign homework in the application. Students need to complete the after-school exercise according to the teacher’s homework requirements, and upload the video of the exercise to the program. Teachers can check the completion of students’ dance movements through the video and give one-to-one guidance. The module of managing and uploading teaching courseware includes viewing and uploading teaching courseware, viewing the published teaching courseware resources and operating the database of teaching courseware. Upload and manage the teaching video module, including viewing the uploaded teaching video, viewing all uploaded video information, shooting time, shooting location, shooting camera model, number of shooting works, video quality, and teaching video database processing.

### Parents’ perception of online dance training

**FIGURE 3:**
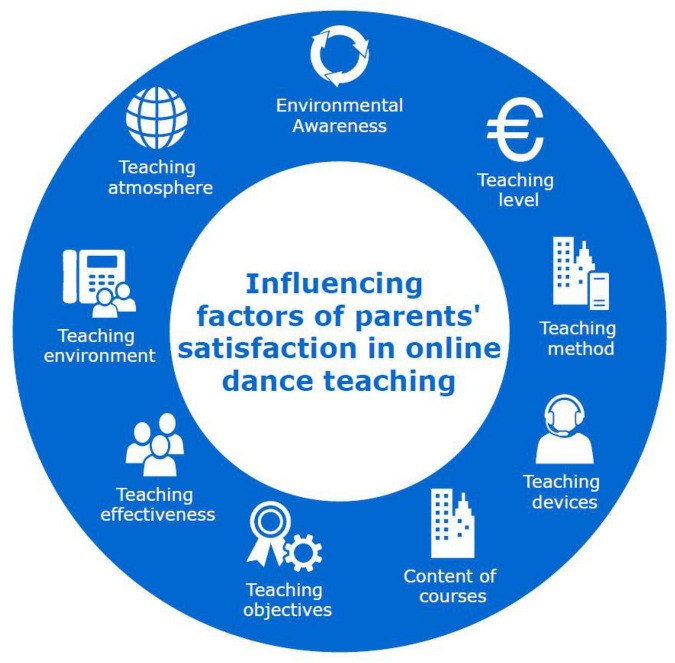
Influencing factors of parents’ satisfaction in online dance teaching.

The teaching goal is the first clear problem in lesson preparation. After all, it determines the teaching content of a class. Teaching methods and the form of teaching organization play a guiding role. Studying teaching materials carefully is the primary basis for determining teaching objectives. Classroom teaching should take the least teaching time to obtain the best teaching effect. We should ensure students’ acquisition of knowledge and development of thinking ability within limited teaching time. This requires teachers to choose the most reasonable teaching methods when preparing lessons, and organize teaching from students’ life experience and existing knowledge background. The factors that parents allow their children to participate in the learning of training institutions are more diverse. Through face-to-face interviews with teachers, parents and managers of training institutions, and telephone interviews with experts, important factors that affect the teaching parents’ satisfaction of children’s sports dance training institutions are selected as comprehensively as possible, and unimportant factors are eliminated. In essence, the process of evaluating online education plans is the process of judging to what extent the courses and teaching plans have achieved the teaching objectives; The purpose of the evaluation is to see to what extent these educational goals have been achieved, and to point out the strengths and weaknesses of the curriculum plan through effective evaluation procedures. Because each indicator is influenced and related to each other. The current online teaching content needs more direct and specific projects, and can provide students with more self-study skills to improve their abilities. Most of the time, basic dance knowledge is an extra-curricular supplement for students. Teachers must pay attention to using more interesting content to attract students’ attention when explaining this knowledge. At ordinary times, teachers should communicate with students more, give some examples for students based on what they have encountered, feel the situation deeply reflected by students, and then use the identity of a person who has come to infect students. In this way, a good and harmonious relationship has been established between students and teachers. Students may also like the teacher and be willing to participate in the teacher’s courses. The measurement scale established in this paper also needs to adjust the correlation of measurement indicators according to the reality and characteristics of online dance training institutions. See Figure 3 for details.

(1) Teaching level
The teaching provided by children’s sports dance training institutions is the content of sports dance, including: acquiring dance knowledge and using dance skills.
(2) Teaching methods and means
Teachers’ teaching method and students’ learning method are the main contents of teaching, both of which are important bases. Without a certain content, the expected teaching goal cannot be achieved.
(3) Teaching content
The content of dance teaching is to convey the main information in the process of using teaching methods and means, including dance theory, dance skills, etc.
(4) Teaching objectives
On the basis of applying teaching level and teaching content, we can achieve teaching objectives. The plan, teaching methods, teaching means, and teaching contents made before the teaching content is delivered serve the completion of teaching objectives.
(5) Teaching effect
Teachers’ teaching level, what teaching methods and means are used, the main teaching contents transmitted, and whether the teaching objectives are finally achieved are the presentation of teaching effects.
(6) Teaching environment and atmosphere
Children’s sports dance training institutions are training sports dance skills, and the training environment of children’s sports dance skills is also a crucial factor in the teaching process, such as the quality of the floor, the placement of mirrors, the number of dance props, and the ventilation of the venue.

In order to integrate learning experiences to form a coherent teaching plan, the process of dividing learning experiences into units, courses and teaching plans. When compiling a set of effectively organized learning experiences, three standards should be met, namely, sequence, continuity, and integration. Customers who often attend training in first-tier cities will have higher expectations for teaching level, teaching content, teaching methods and means; However, a parent from a third-and fourth-tier city doesn’t have high expectations for the teaching methods of educational institutions, so this is also influenced by personal cognitive level. Besides, exaggerated advertising or publicity will also raise parents’ expectations of institutions. For example, the first-class teachers from China Sports Dance Association, etc., parents naturally have very high expectations for the teaching effect provided by them.

## Analysis and discussion of results

The dominant indicator “service quality” is decomposed into three indicators: service attitude, parents’ complaint handling and parents’ rights and interests protection. Then, the decomposed three indicators are compared pairwise to obtain the comparison matrix of the three indicators, as shown in Table 1.

**TABLE 1:** Index comparison matrix.

Quality of service	Service attitude	Parent complaint handling	Protection of parental rights and interests
Service attitude	2	6	4
Parent complaint handling	2/6	2	2/4
Protection of parental rights and interests	2/4	4	2

Then, each column of data is normalized, and a new eigenvector matrix is obtained by dividing the relative coefficient of each column by the sum of elements of each column, as shown in Table 2.

**TABLE 2:** Eigenvector normalization matrix.

The feature vectors are normalized	First column	Second column	The third column
First column	0.651	0.554	0.691
Second column	0.132	0.112	0.076
The third column	0.216	0.334	0.232

Finally, the data of each row is normalized, and the arithmetic mean of the sum of the data of each row is taken to obtain the weights of the three indicators, which are 0.632, 0.105, and 0.259, respectively.

**FIGURE 4:**
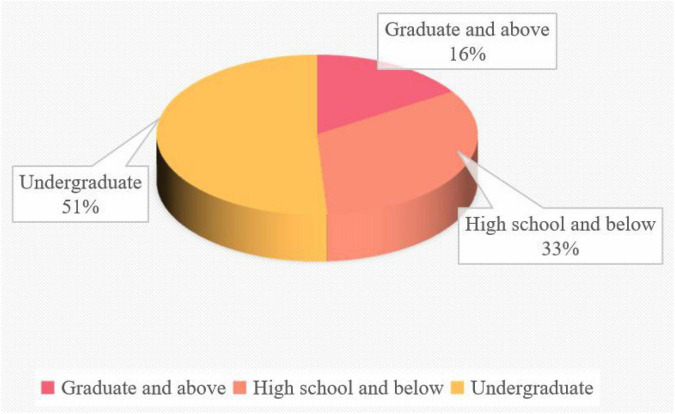
Education level of parents.

As can be seen from Figure 4, among the parents of online dance teaching and training institutions in the sample, bachelor’s degree accounts for the majority, accounting for 51% of the sample, followed by bachelor’s degree or below, accounting for 33% of the sample, and graduate degree or above, accounting for 16% of the sample. It is concluded that the parents of online dance teaching and training institutions in the sample have good educational experience and education background.

**FIGURE 5:**
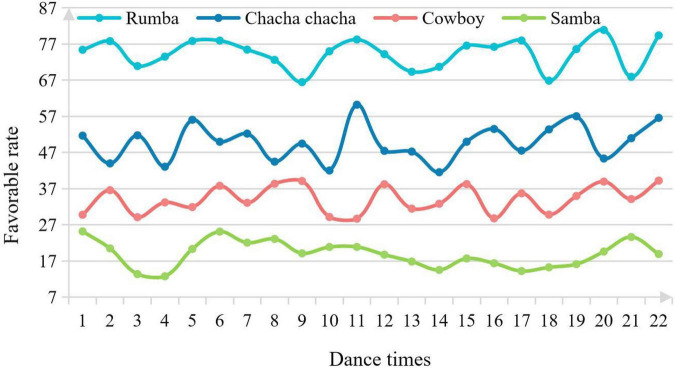
Praise rate of students who have learned dance.

Online dance training institutions can enhance the ability of students in the training service, including physical ability, professional and technical ability and self-confidence ability, according to the different purposes of parents’ choice of projects. At the same time, it is also necessary to properly take into account the acceptability of students at different ages and provide them with interesting and effective training content. In addition, according to the needs of students and their parents’ professional path, training institutions should set up scientific and reasonable advanced learning classes accordingly. Therefore, this experiment adopts the following four dance categories to conduct an experimental investigation on the praise rate: Rumba, Cha Cha Cha, cowboy, and Samba. The experimental results are shown in Figure 5.

Among the majors that children are studying, rumba accounts for the vast majority and is the most popular. The highest praise rate is 81.69%. Chaqia ranks second, with a highest praise rate of 58.35%. Cowboy ranks third, with a highest praise rate of 39.63%. Samba ranks last, with a highest praise rate of 25.03%. It can be seen that most parents prefer to let their children learn Latin dance. It is generally believed that learning standard dance is boring and requires high venues. Among the learned dances, rumba and Cha Cha Cha dances account for the majority, which indicates that they are the most popular dances to some extent. On the other hand, it also shows that the training time of each dance is uneven.

As shown in Table 3, the age composition of parents is mainly concentrated in the two ranges of “31 to 40” and “41 to 50,” accounting for 67.33 and 22.67% of the total sample respectively. The age range of children corresponding to the parents of this age class is the youth mainly accepted by online dance teaching and training institutions.

**TABLE 3:** Age of parents.

Age	Number of persons	Percentage
22–32	35	7%
33–43	302	67.32%
44–50	101	22.66%
Above 50	8	1%

**FIGURE 6:**
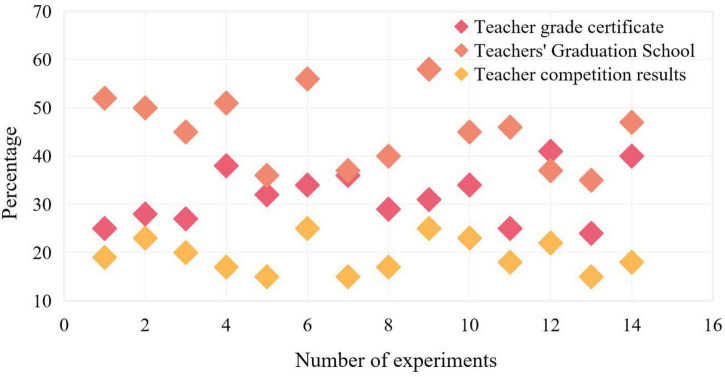
Channels for parents to understand teachers’ professional level.

In this experiment, the professional level of teachers is known through various channels, and the channels for parents to understand the professional level of teachers are studied. The following three categories are used for experimental comparison, namely, the grade certificate of teachers, teachers’ Graduation colleges, and teachers’ competition results. The experimental results are shown in Figure 6.

Among the three categories, teachers’ Graduation schools account for the highest proportion, with an average proportion of 47.07%, followed by teachers’ grade certificates, with a proportion of 32.29%, and teachers’ competition scores of 25.57%. Therefore, in the process of operation, online dance training institutions can display teachers’ relevant professional displays through appropriate ways to meet parents’ needs for understanding teachers’ professional level.

**FIGURE 7:**
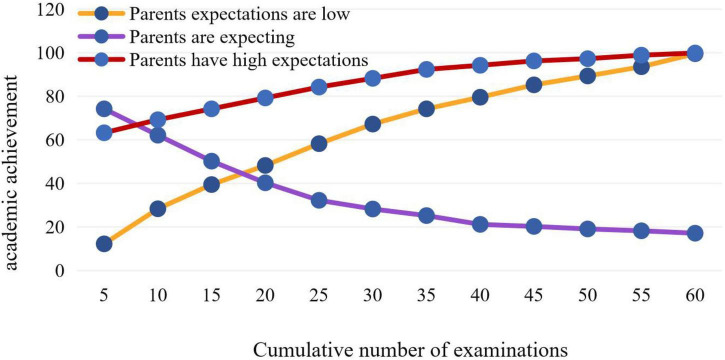
Trend chart of parents’ expectations.

In order to observe this regulation more intuitively, this paper divides the samples into low parental expectation and high parental expectation according to different levels of parental expectation, and draws an interaction diagram of parental expectation, as shown in Figure 7.

When the family’s expectations are low for a long time, with the increase of the number of examinations, the academic performance shows an upward trend. When the parents’ expectations are high, the parents pay high attention to the children and have a lot of communication with the teachers. At this time, the teachers’ expectations and parents’ expectations should promote each other and promote the children’s learning motivation; However, when the family’s long-term expectation is medium, it means that the child does not feel what the parents expect of him. The indifference of the parents’ attitude may make the child indifferent or even resistant to the expectations of the teachers, and then affect the academic performance.

**FIGURE 8:**
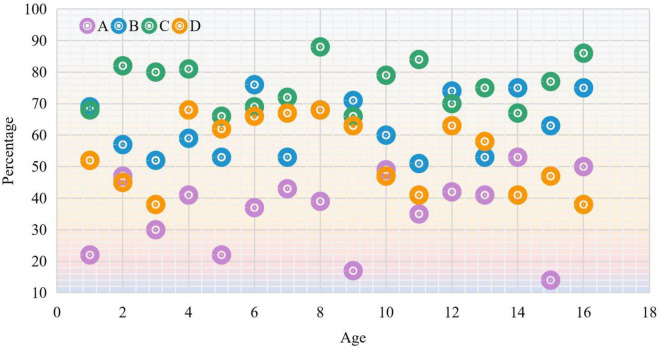
Relationship between reasons for training and age.

Today’s education for teenagers is a comprehensive education, and parents need to spend a lot of energy and money on their children. Therefore, online dance training institutions for teenagers should rely on the local economic situation to develop products with higher cost performance in future courses, activities and other related charging standards. Therefore, this experiment mainly analyzes the reasons for participating in online dance training and the children’s age group, and comes to the following reasons: exercise (a), cultivating children’s self-confidence (b), cultivating children’s temperament (c), and children like (d). Therefore, data statistics are made on the correlation between the above four reasons and age, and the experimental results are shown in Figure 8.

On the statistics of the reasons for participating in the training and the changes between ages, it is believed that online dance teaching and training can cultivate children’s temperament, which is the first reason; the second is that it can cultivate confidence in the second place; then the third one thinks it is possible to exercise, and the last one comes to the training because the children like it. Let the children participate in the training.

**FIGURE 9:**
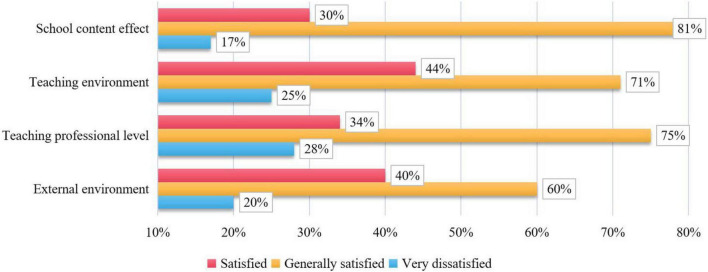
Distribution of perceived quality evaluation.

The quality perceived by customers comes from customers’ feelings. Customers will not use special scientific instruments to measure the quality of products like enterprises, but mainly evaluate the quality of products through vision, hearing, feeling, and the analysis and judgment of the information they know. In addition, subjectivity is also reflected in that different people have different aesthetics and values. For the same product, different customers will have different quality perceptions of the product. This paper analyzes the evaluation of parents’ perceived quality from four index factors: external environment, teaching professional level, teaching environment, and school content effect. According to the average score of the comprehensive evaluation of perceived quality, the differences of perceived quality among parents of different ages, parents of different genders and students of different training times are analyzed. The distribution of perceived quality evaluation is shown in Figure 9.

The average score of the comprehensive evaluation of perceived quality has four index factors, which are: external environment, teaching professional level, teaching environment, and school content effect. The order of satisfaction is: external environment > teaching environment > teaching professional level > school content effect. The purpose of quality analysis is not to rank teachers and students. With the help of an effective quality analysis system, we can deeply mine the basic data, compare horizontally and track the teaching quality vertically. Diagnose and feed back the teaching quality in an all-round and multi angle way, and help download schools to find problems in the teaching process. Help teachers find problems in the teaching process, and help students and parents find problems in the learning process. From a deep level of multi angle and multi-faceted analysis, through exquisite teaching quality analysis and teaching quality tracking analysis, we can find and solve problems in time, so as to achieve the purpose of improving teaching quality.

## Conclusion

The business models of online educational institutions all rely on the technical support of the Internet. No matter what kind of model, it is necessary to connect the platform and users through the Internet. The stable operation of online educational platform is crucial to the development of online educational institutions and has an important impact on customers’ trust in online education. In this paper, the influence of parents’ perception on the brand recognition of online education and training is studied. Through the analysis of online dance training institutions, the research shows that among the three categories, teachers’ graduation colleges account for the highest proportion, with an average ratio of 47.07%, followed by teachers’ grade certificates, with a ratio of 32.29%, and teachers’ competition scores of 25.57%. Therefore, in the course of operation, online dance training institutions can display teachers’ professional displays through appropriate channels to meet the needs of parents for their understanding of teachers’ professional level. Parents have a better understanding of brand recognition through online educational institutions in business model innovation. They should pay attention to independent research and development of new patented technologies and introduce advanced new technologies to assist classroom teaching. For the live broadcast one-to-one mode, class mode and 020 mode, advanced AI and big data technologies can be introduced to assist classroom teaching to attract students’ attention and improve learning efficiency. In the perceived quality, the service has reached the expected level, the internal and external environment of the institution, the degree of brand recognition of learning effect has reached the expected level, and the security score is low. The institution has not done enough for parents’ expectations, and the products and services have room for improvement. In terms of perceived quality, the service has reached the expected level. But the safety score is very low. Therefore, there are certain limitations, which need to be further discussed and analyzed in the future.

## Data availability statement

The original contributions presented in this study are included in the article/supplementary material, further inquiries can be directed to the corresponding author.

## Author contributions

Both authors listed have made a substantial, direct, and intellectual contribution to the work, and approved it for publication.
